# Social and traditional practices and their implications for family planning: a participatory ethnographic study in Renk, South Sudan

**DOI:** 10.1186/s12978-016-0273-2

**Published:** 2017-01-17

**Authors:** Khalifa Elmusharaf, Elaine Byrne, Diarmuid O’Donovan

**Affiliations:** 1 Graduate Entry Medical School, University of Limerick, Limerick, V94 T9PX Ireland; 2 Reproductive & Child Health Research Unit (RCRU), University of Medical Sciences & Technology, Khartoum, Sudan; 3 Royal College of Surgeons in Ireland, Dublin, Ireland; 4 National University of Ireland Galway, Galway, Ireland

**Keywords:** South Sudan, Family planning, Maternal health, Conflict affected fragile states

## Abstract

**Background:**

Understanding what determines family size is crucial for programmes that aim to provide family planning services during and after conflicts. Recent research found that development agents in post conflict settings do not necessarily take time to understand the context adequately, translate their context understanding into programming, or adjust programming in the light of changes. South Sudan, a country that has been suffering from war for almost 50 years, has one of the highest maternal death rates and the lowest contraceptive utilization rates in the world.

**Methods:**

This research used Participatory Ethnographic Evaluation and Research (PEER) to provide a contextualised understanding of social and traditional practices and their implications for family planning. Fourteen women were recruited from 14 villages in Renk County in South Sudan in the period 2010–2012. They were trained to design research instruments, conduct interviews, collect narratives and stories and analyse data to identify, prioritize and address their maternal health concerns.

**Results:**

As a result of wars, people are under pressure to increase their family sizes and thus increase the nation’s population. This is to compensate for the men perished in war and the high child death rates. Large family size is regarded as a national obligation. Women are caught up in a vicious cycle of high fertility and a high rate of child mortality. Determinants of large family size include: 1) Social and cultural practices, 2) Clan lineage and 3) Compensation for loss of family members. Three strategies are used to increase family size: 1) Marry several women, 2) Husbands taking care of women, and 3) Financial stability. Consequences of big families include: 1) Financial burden, 2) Fear of losing children, 3) Borrowing children and 4) Husband shirking responsibility.

**Conclusion:**

The desire to have a big family will remain in South Sudan until families realise that their children will live longer, that their men will not be taken by the war, and that the costs of living will be met. In order to generate demand for family planning in South Sudan, priority should be given first to improve infant and child health.

## Plain english summary

Understanding what determines family size is crucial for planning and implementing reproductive health and family planning services during and after conflicts. Recent research found that organizations working in post conflict settings do not necessarily take time to understand social and traditional practices adequately, translate their understanding into programming, or adjust programming in the light of changes. South Sudan, a country that has been suffering from war for almost 50 years, has one of the highest maternal death rates and the lowest contraceptive utilization rates in the world.

We conducted a participatory research in Renk County in South Sudan in the period 2010–2012 to gain in-depth understanding of social and traditional practices related to family size and its determinants.

We found that large family size is regarded as a national obligation. People are under pressure to increase their family sizes and thus increase the nation’s population. This is to compensate for the men perished in war and the high child death rates. High child death is causing women to have more children, and that in turn is increasing rates of repeated childbearing, maternal and child deaths. The desire to have a big family will remain in South Sudan until families and communities realise that their children will live longer and that their men will not be taken by the war. In order to generate demand for family planning in South Sudan, priority should be given first to improve infant and child health.

## Background

South Sudan suffered from high intensity conflict during the second Sudanese civil war (1983–2005), later on becoming an independent nation as of 9 July 2011. Since December 2013, South Sudan has experienced repeated eruptions of war, violence and political instability.

South Sudan has one of the highest maternal mortality rates worldwide at 2037 deaths per 100,000 live births [1]. Health status indicators have deteriorated over the last five decades and remain among the lowest in the world [2]. Social determinants of health, social rules and values in South Sudan have all been devastated by the conflict [2, 3]. The war exacted enormous destruction, especially on women and children. War and traditional practices have resulted in women in South Sudan having very little control over reproductive decisions, being exposed to sexually transmitted diseases, and having unwanted pregnancies [3]. The war placed high pressure on South Sudanese women to compensate for the high rates of child death and to reproduce as a national obligation [4].

South Sudan has one of the lowest contraceptive utilization rates in the world which is less than 1% [1]. Previous research from South Sudan stated many reasons for this low rate, such as, lack of the availability of contraception, cultural and political barriers [5], illiteracy, and lack of education about reproductive health and family planning [6].

Understanding what determines family size is crucial for planning and implementing maternal and reproductive health programmes. The importance of understanding the cultural, social, economic, political and historical context in addition to understanding demand challenges for maternal healthcare when working in conflict-affected fragile states are well documented [7, 8]. However, many of the health and development projects in South Sudan are significantly hampered because of the mismatch between the understanding of the context by the programme developers and the eligible users of the services. Development agents do not necessarily take the time to understand the context adequately, translate their context understanding into programming, or adjust programming in the light of the context [9, 10]. The research described in this paper is from a study that aimed to gain an in-depth understanding of the social determinants of family size in South Sudan to inform local policy and practice [11].

## Methods

### Study population and setting

This study was conducted in Renk County in South Sudan in the period from 2010 to 2012. Renk county is one of 13 counties that constitute the Upper Nile State. Renk country is located in the northern part of the state, close to the international border with the Republic of Sudan. The administrative capital of Renk county is Renk town, which lies on the eastern bank of the White Nile. Renk county is sub-divided into four political subdivisions or ‘payams’: Renk town, Geigar, Shomedi and Galhak. Each payam consists of several ‘bomas’ which are the smallest administrative units.

South Sudan’s largest oil fields are located in the Upper Nile state, around Renk county: about 77% of the estimated remaining commercial reserves of South Sudan’s oil are located in the Upper Nile state [12, 13]. Despite the number of oil drilling sites in the Upper Nile State, the state remains extremely poor, with limited public services and basic development indicators [14]. More than 90% of the population in Renk district live on less than USD $1 a day [15]. The state is characterised by underdevelopment, lack of infrastructure, harsh environmental conditions and an influx of large numbers of returnees.

As is common in socially deprived situations women and children often suffer the most. Traditionally women are considered to be responsible for domestic concerns, and for providing for their families. They are often abused when carrying out these roles. Whenever husbands are unable to provide the necessary essentials for the welfare of his family, the frequency of domestic violence, mainly wife beating, rises significantly. Violence against women occurs frequently, but gets little attention [16, 17].

**Fig. 1:**
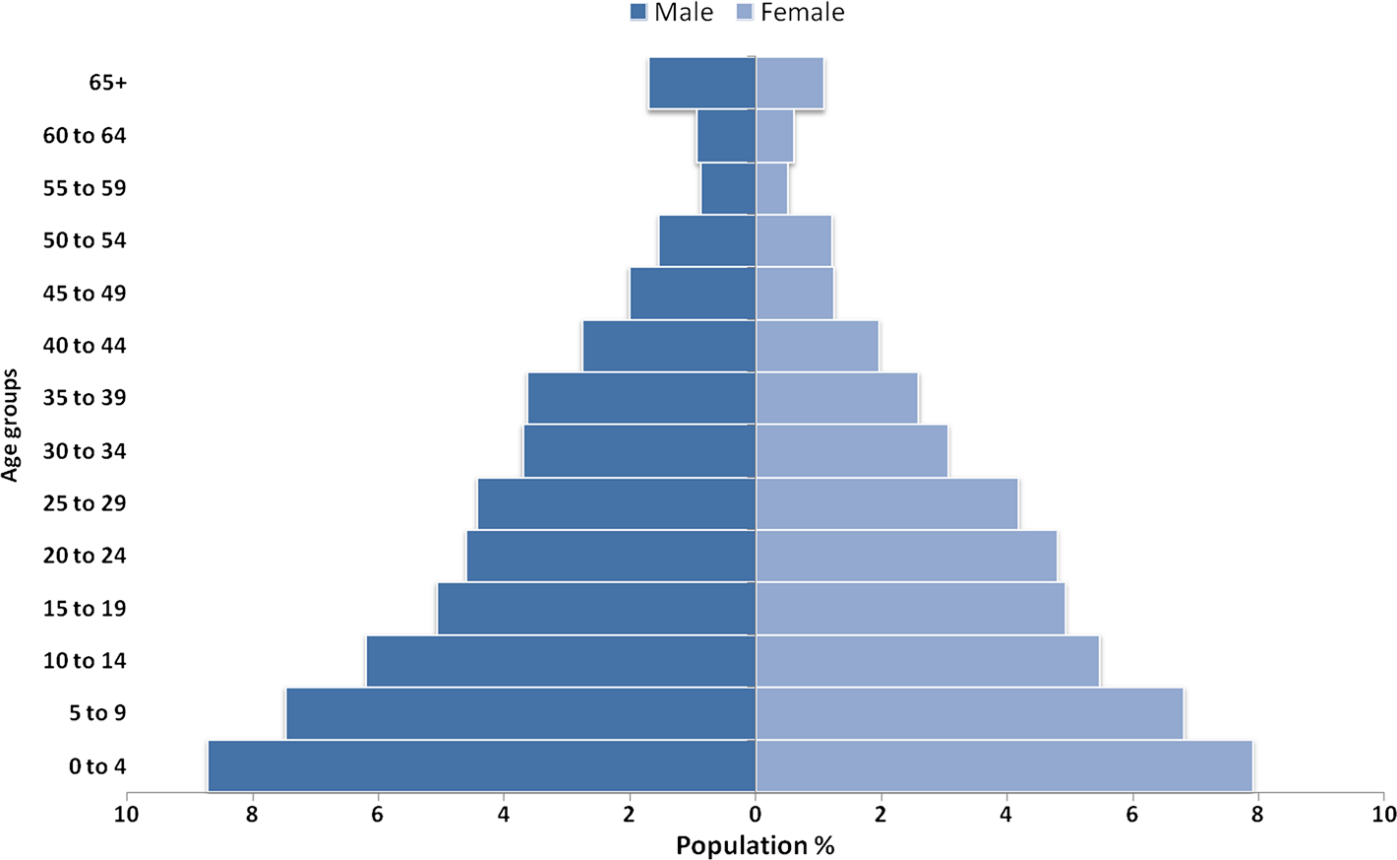
Population pyramid for Renk County (2008) Source: Census figures of April 2008 [18]

Renk county is well known for the diversity of its population, mixed identity, generations of intermarriage and cultural exchanges between different parts of Sudan. The nature of this diversity is derived from the fact that Renk has numerous cross-border activities, which attract traders from other counties. The people of Renk county are predominantly Nilotics, and the majority are members of the Dinka ethnic group. The remainder of the population are from the Shilluk, Nuer and other non-Southern tribes such as Dago, Burun, Funj and Selaim. In the April 2010 census, the population of Renk County was 137,751, in an area of 10,031 km^2^ (population density 13.7 per square kilometres) (Table 1 and Fig. 1). The male to female ratio was 1:1.16. The number of households was 24,206, with an average household size of 8.5 members, ranging from two to 19 members [18, 19].

**Table 1:** Populations by Payam, Renk county

Payams	Total Population	Male	Female	Number of Households
Renk	69,079	36,790	32,289	11,684
Geger	39,649	20,620	19,029	7234
Jalhak	17,436	9920	7516	3093
Shomedi	11,587	6639	4948	2195
Total in Renk County	137,751	73,969	63,782	24,206

Source: Census figures of April 2008 [18]

This study was conducted during the South Sudan referendum, which witnessed the return of many people to Renk. A survey in 2012 found that between 2010 and 2012 the percentage of returnees to Upper Nile State had increased by 10%, and more than 20% of households comprised returnees [19]. Most of the population of Renk county is very young, with 30% of the population under 10 years, and 70% of the population under 30 years. The population of women of reproductive age (15–49 years) in Renk county is 29,589. This population pyramid of the Renk county population indicates a high birth rate, a high death rate and a short life expectancy [18].

Renk county, though having large oil reserves, has extremely high maternal and infant mortality, poor social and weak economic conditions. The effects of war remains and is evidenced in the poor infrastructure, the mental health of returning soldiers and refugees, and the violent society in which women and children live.

### Study design

This study used a participatory research methodology known as Participatory Ethnographic Evaluation and Research (PEER) to provide a contextualised understanding of determinants of family size [20, 21]. PEER’s main tenet is to work with local women - who already have established trusted relationships with the people who are the focus of the research – to collect contextualized data under conditions in which participants in conflict affected zones might not have the most trust of researchers. For this study, illiterate marginalised women in Renk County were trained to design research instruments, collect and analyse qualitative data in forms of acting dramas and developing profile stories. This approach was used to generate an in-depth and contextual understanding of reproductive health. PEER has been used in reproductive health research in many settings [22].

### Sampling and participants

Representatives of a local NGO, the Director of Preventative Medicine, the Director of Reproductive Health and the Midwifery School at the State Ministry of Health, and the vice-governor of Renk County were involved in the research from the outset and helped to recruite local women for this research project. The criteria for recruiting the women and why these criteria were needed was agreed in the meetings. Women were recruited through traditional village leaders. Sixteen women from 16 villages were initially nominated and enrolled. Two dropped out - they did not attend after the first workshop. The women attended a 4-day participatory training workshop to develop their research skills: designing research instruments, conducting interviews, collecting narratives and stories and analysing data. During the workshop, the women discussed important maternal health issues in their community, and identified key themes and questions for the qualitative research. As the women were illiterate, the interview guidelines comprised some images they developed to remind them of the questions to be asked.

### Data collection

Women returned to their villages to carry out in-depth interviews in their local language with three of their friends over 3 weeks. The principle researcher visited them in their homes to collect their findings in a series of debriefing sessions. the principal researcher debriefed the PEER researchers in the Juba Arabic language. This ‘pidgin’ language is derived from Sudanese Arabic that people from both North Sudan (where the principle researcher comes from) and South Sudan can speak and understand [23]. Upon completion of the interviews with peers and the debriefing sessions, the women came together with researchers, for a second workshop. During the second workshop, the main themes from the interviews emerged. To disseminate the findings and address some of the inaccurate knowledge and practices, dramas and profile stories were developed.

### Data analysis

Thematic data analysis was conducted jointly with the PEER trained women and the research team. Data included in the analysis were the debriefing transcriptions, the workshop discussions, findings and outputs. The research team employed an inductive approach to ensure that the identified themes were data-driven and strongly linked to the data, without trying to fit it into a pre-existing coding frame. The entire data set was coded using NVivo 10 from which the major themes were emerged by merging, renaming or making ‘parent–child’ relationships, and trying to identify relationships between each such pair. The researchers reduced overlap between codes, sorted the remaining codes into potential themes and organised all the relevant coded data extracts within the identified themes. How emerging themes relate to each other was then be explored. Ultimately the ‘defined and refined’ themes and sub-themes were organised into a coherent and consistent account with an accompanying narrative. Overall, the five phases suggested by Braun and Clarke [24] for thematic analysis were conducted collectively: data management and familiarisation, initial coding, identification of themes, reviewing themes and defining and naming themes.

### Ethics

The research followed an informed consent procedure consistent with international standards and appropriate to the research context. Considering that this research was conducted in a conflict affected fragile state, the authors decided on using verbal consent without documentation due to two factors: 1) The majority of participants are illiterate and unaccustomed to dealing with forms, and 2) the consent document will be the record linking the subject and the research and the principal risk would be potential harm resulting from a breach of confidentiality. However, all reasonable steps were taken to ensure that participants were informed that they could collaborate freely and without coercion. The principle researcher described clearly the research and the role of the participant, the commitment involved, reasonably foreseeable risks and expected benefits. The principle researcher explained to the participants how information that may identify individuals or communities is managed, including the extent to which confidentiality and/or anonymity is guaranteed. Contact details of the research team were given to all participants with the information that they can contact them should they have any questions or concerns. Throughout the research process it was emphasised that participation is voluntary, that participants have a right to withdraw at any time and that no sanctions will be imposed for either non-participation or withdrawal. Participants were asked permission to record the interview. When they denied permission the recorder was not used. The costs borne by participants such as the bus journey and their telephone calls were compensated. Food and accommodation were provided to participants for the duration of the workshop. No monetary incentives were provided.

## Results

**Fig. 2:**
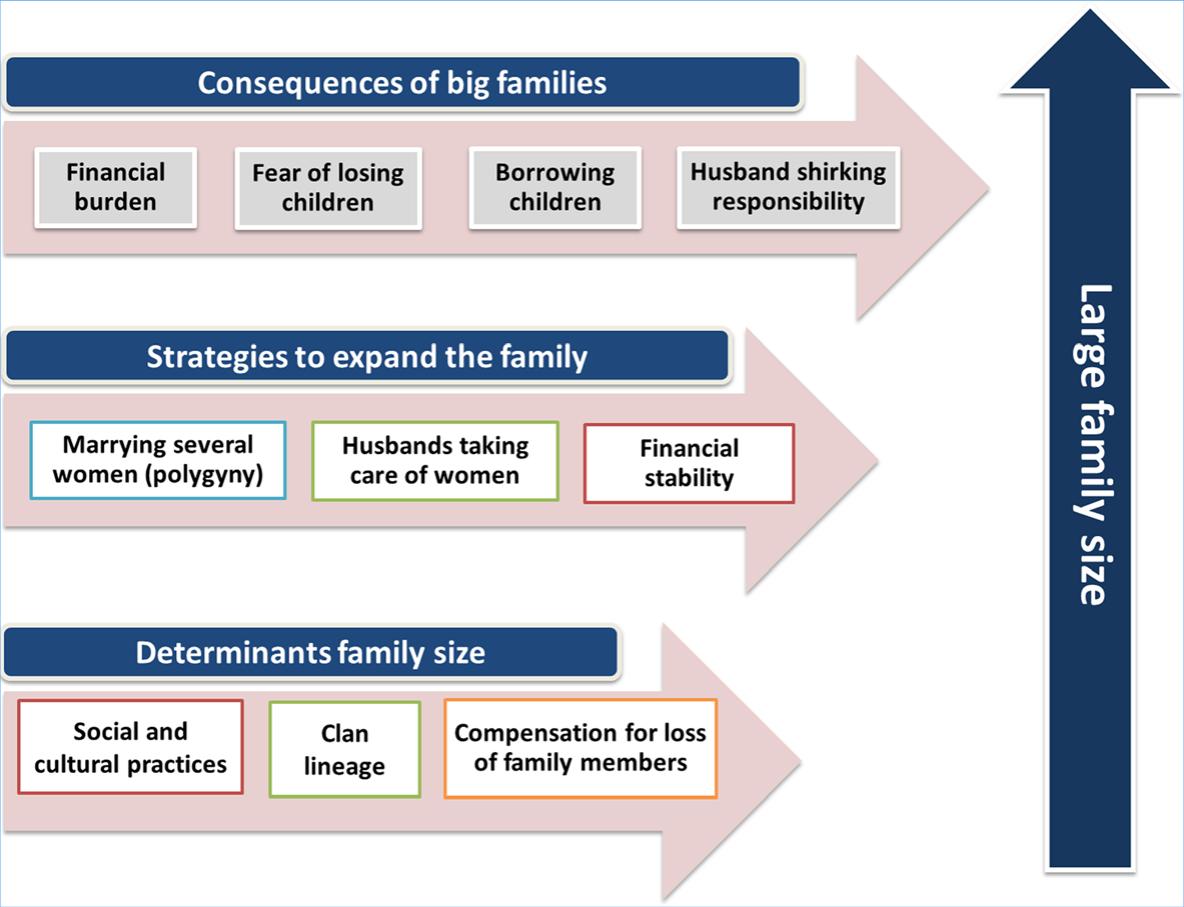
Thematic framework for the social and traditional practices and implications for family planning

As shown in Fig. 2, three main themes emerged from the data: 1) Determinants of family size, 2) strategies to expand the family and 3) consequences of big families.

### Determinants of family size

#### Social and cultural practices

There is huge social pressure on couples to have children in Renk. Marriage is considered incomplete until a child has been born. The real security and stabiliser to the indissolubility of marriage lies in having children. The husband and wife are both equally anxious for a child to be born. They and their families worry about infertility. A woman who does not conceive is considered shameful. Husbands are admonished for not being able to make their wives pregnant. Husbands are concerned about seeking treatment or marrying another wife if they fail to have children, as pregnancy brings happiness to him:

*If a lady doesn’t bring a child that means she’s not good and people don’t like her. (R11)*

*The woman should marry to bring children that will benefit her later, if she doesn’t have children her heart will not rest. Her tummy brings six or seven children. The mother and father will not say we don’t want children, no one refuses to have a baby, but it is God’s wish. (R6)*

*In our traditions, it’s disgraceful if a woman still gets her periods and doesn’t become pregnant. People quarrel with him – why don’t you make the woman pregnant? (R7)*

Childbirth is considered a return on investment. That is way people celebrate the birth of a new born. As part of the celebration, grandparents provide cows, furniture, groceries, oil and alcohol. People also celebrate by holding a feast, partying and dancing.

*The family who paid the dowry will be happy when the woman gets pregnant. They won’t feel like they lost money over her. The family will be happy because a new baby is born and it will be an addition to the family. The whole family will be very happy and they will party (nuggara) and dance. (R10)*

Social cohesion is deeply rooted in the culture of South Sudan. People advise and support each other. They face and solve problems together. Large family size plays an important role in consolidating social cohesion which is strengthened by the presence of multiple supportive social networks of families, friends, neighbours, heads of tribes and local tradesmen:

*The big family is good. People agree and do one thing; if they want to do anything you all get together and help each other. If they all going to harvest on the farm they will all get together. (R13)*

*If there is a problem the family will face it. For example, if a son made a problem, they bring him and say whose child is this. The family is the one that stands up to face the problems and solve it. If the family have same points of view, then they can improve their family. (R10)*

#### Clan lineage

The grandfather looks for a child to carry his name. He brings cows for the “naming ceremony” where family, friends and neighbours will be invited for food and drinks.

*The husband’s father when knows that the woman get pregnant, he knows that the child will raise the family name. They will name him after his grandfathers’ names, so the name will not disappear. (R6)*

Creating big families is an essential concept to sustain the name of the family and to have more social influence and status. Participants stated that ‘khashm al-bayt’ (mouth of the house) is very important; this term refers to a collection of big families that descend from a common ancestor. ‘Mouth of the house’ is the building block of a clan, and a clan is the building block of a tribe:

*If the children were many they will improve the family, and they will be ‘khashm al-bayt’ (the mouth of the house). The mouth of the house means if people come and visit us they will say this is the family of X. The children when grown up they will marry and have children again and make a family. … If they grow up and get married they will be ‘the mouth of the house’. Nothing is as important as the children. (R3)*

*They are proud when they have lots of children and then people can say that person has lots of children. Children get older and carry your name. If the father dies, the family’s name would remain, the family would continue. (R5)*

#### Compensation for loss of family members

The loss of children due to death during birth or disease is a major concern. The family considers the death of a child as ‘*khasara*’ (a waste). Participants were aware that nutrition and sanitation were important for child health, but death of babies were common. According to participants, three to four out of every 10 children are likely to die. Participants were aware of the importance of a family taking care of its children and taking them to the hospital when the children are sick – if not, families loose and have fewer children. Due to the unpredictable nature of determining the number of children surviving until adulthood, families choose to have large number of children.

*Some families have six or eight children, because if you get a lot, half of them might live and half might die. If four die, then four will be alive. (R7)*

There is also concern that some children might not be socially successful or might be difficult for their parents to control. Therefore, parents try to have many children to compensate for those ‘bad children’:

*The man can bring many kids so he can send them to school. Maybe one will be a doctor, one will be a teacher and of course some others might stay without any work. If you bring kids, say seven, maybe three out of them will be not good in education, and you don’t benefit from them. So that’s why having many children is very important. (R12)*

People believe that the number of children a couple have is in God’s hands. They accept the death of children as a normal phenomenon, and believe that children will only live if God spares them:

*If God wants her to have lots of children, then there will be lots of children in the family. (R10)*

*God takes some, and leaves some. It is not guaranteed who is going to live or die. (R7)*

The long civil war in South Sudan killed many men, leaving many widows. Many women have few children because their husbands went to war and never came back:

*Most men were taken by the army for the war, that’s why women are left with no husbands. She can have only two children, but lots of men were killed during the war so that’s why women don’t get pregnant. (R7)*

The years of war and conflict, as well as the post-conflict period, resulted in fear and instability. People still continue to be concerned about protecting their property, preparing for upcoming unpredicted war or fights, and re-establishing families and clans – in effect compensating for the population and family members lost in the war.

*In the war, lots of men died. Some families have completely disappeared because of the war in the south. That is why men now marry four wives who could all get pregnant in the same year and he can have four new babies in 1 year. (R7)*

Most of the fighting and conflict in the area is related to farming and lands. People are worried about attacks on their arable land and believe that the family should be large and strong, so they can fight, protect and defend themselves:

*We live where Denka tribe live, if you have a farm and it has borders, someone from outside can’t take your farm if he sees that your family is big. One person cannot defend against an outsider by himself, but when a family is big whoever wants to bring something bad cannot because they are many. (R1)*

*Here, sometimes people fight or go to war. Men marry more than one wife to create a large family. Each woman might have five to 10 children, so they have large families. If war arises, they can attack the other one. But if you have just one woman, you might have only one child, so you can’t fight. (R12)*

### Strategies to expand the family

#### Marrying several women (polygyny)

Polygyny (a narrow form of polygamy) is the practice of having more than one wife at one time. Participants reported that a woman’s worth is only to give birth and raise the children. The man sees the woman as his property, having ‘bought’ her with cows. It is common that a man can be married concurrently to more than one woman. If a man is able to afford the high bride price in the form of cows, he has the right to marry again.

*Families here consist of a mother, father, children and two, three or even four wives to bring lots of kids. There is one father but many wives; there is no trouble because a man who is strong solves his family’s problems. A man who has cows, goats or money can raise his kids and marry more than one woman. (R1)*

If a woman gets married and does not get pregnant, she may be considered useless, and the husband will find another woman to marry:

*If the woman didn’t get pregnant, they will take her to seek treatment to bring children. If she didn’t get treated, the husband will marry another one. (R6)*

Households were men have more than one wife are housed within one big yard with multiple huts where each wife lives separately, taking turns to prepare meals but they eat together. The participants noted that while jealousy does arise for some, the majority do not have a problem with these arrangements, seeing benefits from living in one place with other wives. The wives must respect each other; otherwise the husband will beat them. If a woman is ‘wrongfully beaten’ she will complain to her father-in-law, who will discipline his son.

Some participants contradicted this, by saying nowadays women are seen as an important part of the household and that the average number of wives has decreased. However, it was also noted that some women try to have as many children as possible so her husband will not have an excuse to marry another woman:

*My husband is a priest in the church and he only has one wife, me. I have six children now. I love him and so I’m prepared to have more children, maybe twelve (laughs). (R7)*

#### Husbands taking care of women

Within the context of difficult life, stress, the multiple responsibilities of women and financial constraints, it is becoming more acceptable for a husband to be seen to take care of his wife, provide her peace of mind, so as to improve her health, thereby enabling her to have more children. There is a belief that rest, relaxation, comfort and avoidance of tiredness and heavy work, enable women to have more children - rest meaning that pregnant women should be able to rest when they need to and only take care of children and the home. A good relationship between a woman and her husband is considered important, because agreement, happiness, honesty and harmony make them bring many children:

*If her husband makes the woman comfortable, takes care of her, looks after her when she’s sick and takes her to the hospital, she’ll bring many kids. My friend told me a story about a woman who gives birth every year, she has lots of things, and her house is clean. Her husband takes her and the kids to the hospital even if she just has a headache. (R1)*

*If you are at home in the shade taking care of your children and the man goes bring water, wood and food you’ll have no concerns or worries, just relaxed at home, your heart relaxed you don’t have any problem. The relaxation and husband not fighting will make you brings you many children. (R11)*

As one interviewee put it, ‘disease takes people backwards’ and so a mother, if ill, should seek treatment so that she can have children. A woman treated for illness is considered a clean woman, ready for having children:

*If the woman got married and has a disease in her tummy and it is not treated she will have few children, but if treated and cleaned she will have ten or six. Don’t let disease stay in your body. If they get sick, there will not be a family. Take care of yourself because diseases take people backwards. (R6)*

*If the mother is healthy, if you take care of the mother and the children properly, this makes you have lots of kids. The woman is healthy when her husband and the doctor take care of her. (R13)*

#### Financial stability

Financial security is considered one of the major determinants of having many children. The ability to provide food and medicine for both mother and children, and thereby meeting the basic needs of life is believed to make women comfortable and relaxed and thereby able to have more children. Women stated that they dream to live in a better place, but because of their illiteracy and poverty their dreams will never come true:

*If the family is (financially) comfortable, and the father is able to provide the wife medicines, in this situation woman can have lots of children. It depends on the father’s (financial) abilities, if people help each other and if they take care of the mother and treat her that makes people bring more children. (R9)*

### Consequences of big families

#### Financial burden

Having many children brings a greater financial burden to a family. The father has to work and provide for his children so that they can go to school. Fathers are concerned about raising their children well, so that they will grow up and do the same for their children:

*You can bring many children, but you have to support and watch them. The mother and father may get tired, but you will be able to raise them well. Then if God willing, even if one rose well, he will support his father and mother. (R6)*

*After he brings the child he raises him and then another child comes after him. If you raise them well then your child will also raise his children well. (R6)*

*You need to take care of everything: the money, raising the children up, studying with them and doing all his duties. The father goes farming so he can bring bread for his children, guides towards the future so they won’t do anything wrong. (R10)*

#### Fear of losing children

Many people who have many children are afraid of the ‘evil eye’. People don’t like to talk about how many wives or children they have as they believe that if people saw their ‘many children’, without saying ‘mashallah’ (God has willed it) or ‘tabarakallah’ (blessings of God), injury and bad luck for the children will follow:

*There is a woman who had a lot of children, these children always play in front of the house, and when the man comes back from work and finds them playing, he shouts at them to go inside. He fights with his wife because she let them play in front of the people’s eyes. He is afraid from ‘oyown al nas’ [people’s evil eyes] (R9)*

*There is a man dividing his house into two yards for his two wives because he said people don’t say the word ‘mashallah’ (God has willed it), that’s why if any stranger come to their house he always makes sure who is he to make sure that his children are safe. (R9)*

#### Borrowing children

Families with many children often give a child to an aunt or uncle who does not have children, so that they can raise the child as their own. Sometimes the aunt or the grandmother borrows a child to help her in the house when she is sick:

*Her sister had no children so she gave her a child to help her in the house and he became like hers. For example, if someone has a child and he dies, his brother gives him one to rise until he grows up and gets married. (R10)*

*These children are not just yours; your aunt or grandmother who is sick or needs help may come to you and ask you to give them a child to help them in the house. (R14)*

*If the mother has no children other than the one who got married then she might ask him to give her one of his children who are between 8 and 10 years old and then this mother will become responsible from this child, and if the child want to go back to his family they will let him go. This was more common in the past. (R14)*

#### Husband shirking responsibility

Examples were given of men leaving their family due to the high level of responsibility placed on the man when there is a large family. In such cases the wife has to stay with her father-in-law especially if she has a child because ‘she married with cows’, and the father-in-law might take the child away from her if she leaves. If she has no children, she still cannot leave because of the dowry of cows, and she will be given to one of his brothers to marry. The husband’s father will gather his sons and tell them ‘this woman is a good woman she is a man’s woman, she stayed and did not leave so I will give her to one of you’. If the woman is strong enough, she can refuse to go to one of her husband’s brothers and wait for her husband to come back:

*There is a lady who has kids, lots of kids. And her husband takes care of her. The man stays with her; he stays for some time and then goes away. So sometimes he stays with her and sometimes he runs away. When he finds it’s difficult to take care of them, he goes away. He goes far away. He stays away 1 or 2 years. When he finds a job, he comes back (R13)*

*If the father is not around, the mother can study with her children and fill the gap. She shouldn’t just say I am only a woman so I can’t teach my children. I know a woman after her husband got married; he left home and left her with five children. Her husband’s family shunned her. This woman started collecting wood and selling it and her children started going to school. Then she started doing business. When her children started secondary school she moved to Khartoum and started doing washing. Now one of her boys is a doctor in Juba and one is a teacher here in Renk. Everybody here wants to be like them. If your husband has died or is in the war you have to do as much as you can. (R10)*

These factors affecting family size, the strategies employed to maintain large families and the consequences of large family size on families has implications for the planning, and delivery of reproductive and maternal health programmes.

## Discussion

The determinants of family size described above are not unique to Renk County or South Sudan. In sub-Saharan Africa, poverty, illiteracy and child mortality have been reported as the main determinants of large family size [25]. Family size is also influenced by cultural factors, such as children’s economic value, and the importance of helping elderly parents, posterity and family lineage [26].

Many studies have shown a positive relationship between child mortality and the desire to have more children [27, 28]. Three mechanisms have been suggested. The first is known as ‘the physiological effect’ in which infant death results in a sudden termination of breastfeeding, resumption of ovulation and an increased chance of getting pregnant sooner [29]. The second one is known as ‘the replacement effect’ in which parents try to ‘replace’ the dead child with a new child, in order to attain the desired number of surviving children [30]. The third mechanism is known as ‘insurance effect’, by which parents try to bear more children in order to protect themselves against any future child death, even if none of the children born ever die [31]. The findings in this study support the replacement effect and insurance effect that underpin high infant mortality rates. Additionally, as a result of war, the people in South Sudan are under pressure to reproduce in order to compensate for the population and family members lost during the war – the former coming from the state as a national obligation [4] and the later coming from within the clan.

The implications for maternal and reproductive health in South Sudan is that the desire to have a big family will remain in Renk county and other parts of South Sudan until families and communities experience their children living longer, and are reassured that their children will not be taken by war. Hence, family planning is not considered an option. Family planning is not culturally acceptable, as the role of women in South Sudan is seen mainly as the provider of children. There is a strong social need to have many children, in order to meet family responsibilities and to be the main family asset, though some women may prevent pregnancy through abstinence.

Consequently, women in South Sudan are caught in a vicious cycle of high fertility, high rate of child mortality, reinforcing high fertility rates and resultant high maternal morbidity and mortality. The fertility–infant/child mortality link is culturally strong and keeps demand for contraception at a very low level. These findings reinforce the conventional demographic theory regarding the early stages of the demographic fertility transition. Large family size is a result of high fertility, and the desire for many children is caused by the need for: children to help with family enterprises; security in old age; and protection against loss or the recovery of past losses [32]. It also reflects the distinctive pronatalist features of African societies, whereby reproduction is promoted for social reasons and to ensure national continuance [33].

## Conclusions

Understanding the social and cultural context of South Sudan should be the key consideration in implementing interventions that can increase access to family planning services. Working at the community level and gaining an in-depth understanding of the local needs, and social and cultural behaviours are essential to understand the context of hard-to-reach communities. By doing this, appropriate services and interventions can be tailored to fit the community and will, therefore, be more likely to be accepted and utilised by them. This requires awareness of health personnel and key stakeholders who will support interventions and address challenges that they will need to overcome. Involving the male partner, empowering women and encouraging stakeholders to focus on demand-side barriers will create demand for healthcare services and respect for their rights. In order to generate demand for family planning in South Sudan, priority should be given first to improve infant and child health.

## Abbreviation

NGO: Non-Governmental Organization
PEER: Participatory Ethnographic Evaluation and Research
